# Graphene Nanopore Arrays for Electron Focusing and Antifocusing

**DOI:** 10.3390/nano12030529

**Published:** 2022-02-03

**Authors:** Damir Mladenovic, Daniela Dragoman

**Affiliations:** 1 National Institute for Research and Development in Microtechnology (IMT), Str. Erou Iancu Nicolae 126A, 077190 Voluntari, Romania; damir.mladenovic@imt.ro; 2 Physics Faculty, University of Bucharest, P.O. Box MG-11, 077125 Bucharest-Magurele, Romania; 3 Academy of Romanian Scientists, 3 Ilfov, 050044 Bucharest, Romania

**Keywords:** graphene nanoribbon, nanopores, electron lens

## Abstract

We have shown, via numerical simulations, that a symmetric array of nanopores with appropriately designed shapes and sizes arranged along an arc of a circle in a graphene nanoribbon can focus or antifocus an incident ballistic electron wavefunction. The position of the focal/antifocal region depends on the electron energy. This effect, which takes place in the energy interval of one-transverse-mode propagation in the nanoribbon, highlights the similarities with plasmonic focusing by an array of holes in a metallic sheet, while emphasizing the differences between the propagation and excitation of electrons and electromagnetic fields. In particular, the electronic antilens has no counterpart in classical optics.

## 1. Introduction

Quantum-classical analogy has proven itself to be a very fruitful research area in physics, leading to the discovery of new phenomena, applications and devices [1]. In particular, the analogy between the propagation of ballistic charge carriers in common semiconductors or graphene, on one hand, and the evolution of classical electromagnetic waves, on the other hand, has enriched both areas of research, leading to the development of domains such as electron optics, photonic bandgap structures, optical Bloch-like oscillations, and spin-related phenomena in optics (see, for instance, refs.[1,2,3,4,5,6,7,8,9,10,11,12,13,14,15] and the references therein). This analogy is based on the formal similarity between the Helmholtz equation for electromagnetic fields and the time-independent Schrödinger equation for charge carriers in common semiconductors and, respectively, the Dirac-like equation in graphene, obtained in the continuum limit as a low-energy approximation of the tight-binding Hamiltonian [16].

Based on the above-mentioned analogy, ballistic-electron lenses were fabricated using electrostatic gating or applying magnetic fields [1,5,15,17,18,19,20,21]. On the other hand, a simple array of holes in a metallic substrate was shown to focus surface plasmon polariton waves [22]. Such a focusing configuration could also be of interest for graphene, since arrays of nanoholes in this material can be fabricated quite easily using standard electron-beam lithography [23,24,25] or other methods, such as microphase-separated block copolymer self-assembly [26]; arrays with sub−10 nm neck widths have been fabricated using the last method. Therefore, the aim of this paper is to investigate if an array of properly shaped and placed nanopores in graphene can focus the wavefunction of charge carriers in the collision-free, ballistic regime. From a practical point of view, electron focusing could be of interest, for instance, in maximizing the connection efficiency between different parts of a nanodevice. This study cannot benefit directly from the well-established ballistic electrons–classical optical fields analogy since (i) the above-mentioned analogy has not involved surface plasmon polaritons to date [27,28], and (ii) the Dirac-like equation does not accommodate defects in graphene, such as nanopores, at least not in a straightforward manner. As a result, numerical simulation methods are essential in answering the question of the possibility of focusing electrons in graphene by an array of nanopores. The results obtained in this paper, which show that not only focusing of charge carriers but also antifocusing could occur, depending on the shape and hydrogenation of pore edges, emphasize the richness of new phenomena that could be evidenced using not only the similarities but also the differences between quantum and classical physics. In this way, our study generalizes the previously mentioned analogies.

## 2. Materials and Methods

We studied wide graphene nanoribbons (GNRs) with armchair edges, containing nanopore arrays with different shapes arranged along an arc of a circle. In all cases, the electronic transport in GNRs was simulated using the quantum transmitting boundary method (QTBM) approach [29,30] incorporated in the multipurpose freeware NEMO5 Parallel Multiscale Nanoelectronics Modeling [31,32,33], which is a better alternative for fast simulations of two-dimensional structures [29,30,34,35,36] than the non-equilibrium Green’s function or the recursive Green’s function methods. Hydrogen passivation of GNR edges and all nanopores, present in realistic structures, was included in our calculations via the three-orbital per atom, nearest-neighbor p/d tight-binding model [37]. The wavefunction distribution for the *Pz* orbital in GNR was obtained by solving the discretized time-independent Schrödinger’s equation for current-carrying states using open boundary conditions [29]. More precisely, using the NEMO5 software, the wavefunction Φ was obtained as a solution of the equation

(1) $$\left(EI\;-\;H\;-\;\Sigma \right)\Phi \;=S$$

where *E* is the energy of charge carriers, *I* the identity matrix, *H* the device Hamiltonian, Σ the self-energy of the contacts and *S* a carrier injection term from the contacts. The Σ term, describing the charge injection and extraction at the two contacts, expresses the open boundary conditions, while the *S* term is associated with the propagating modes of the translational-invariant contacts. In the QTBM simulation algorithm [34], the self-energies of the contacts are first solved using a transfer-matrix method, and then the wavefunction over the entire structure is obtained as the solution of the linear Equation (1) in a computation time that is independent of the potential energy landscape in the device. Once Φ is known, other observables associated with the transport of charge carriers in a general device with multiple input and output leads, such as transmission and density of states, can be calculated. 

It should be mentioned that NEMO5 relaxes the structure to its lowest energy, prior to charge transport calculations, determining the hydrogenation type in the process: with one or two H atoms linked to C atoms at the boundary. In particular, the GNR sides are terminated/passivated with one H atom. Note that both hydrogenation types are stable under certain conditions pertaining to the chemical potential [38], and are known to influence the band structure of GNRs, in particular of armchair GNRs [39]. Double-hydrogenated zig-zag edges of triangular graphene nanoflakes have been recently observed experimentally and it was shown that one of the two passivating hydrogen atoms can be successfully removed by ramping up the bias of a scanning tunneling microscope tip [40].

Because light localization in the plasmonic lens is due to the interference of waves originating at the pores [22], we expected that electron localization would also be a result of the wavefunction interference pattern. Thus, in order to achieve the maximum effect, we chose, in all cases studied in this paper, the electron energy *E* in the range of single-transverse-mode propagation. This range is determined by the width *w* of the rectangular GNR, which is continued at the left and right sides with infinite graphene leads of the same width *w*. Throughout the paper, we assumed that electrons propagate from the left to the right side of the structure, of length *l*. Note that the size of the rectangular GNR was limited to around $2.7\times 10^{6}$ atoms by the heavy workload and memory constraints of the simulation hardware; in this case, the time needed to complete the simulation per electron energy value was around $5\times 10^{4}$ s.

## 3. Results

**Figure 1:**
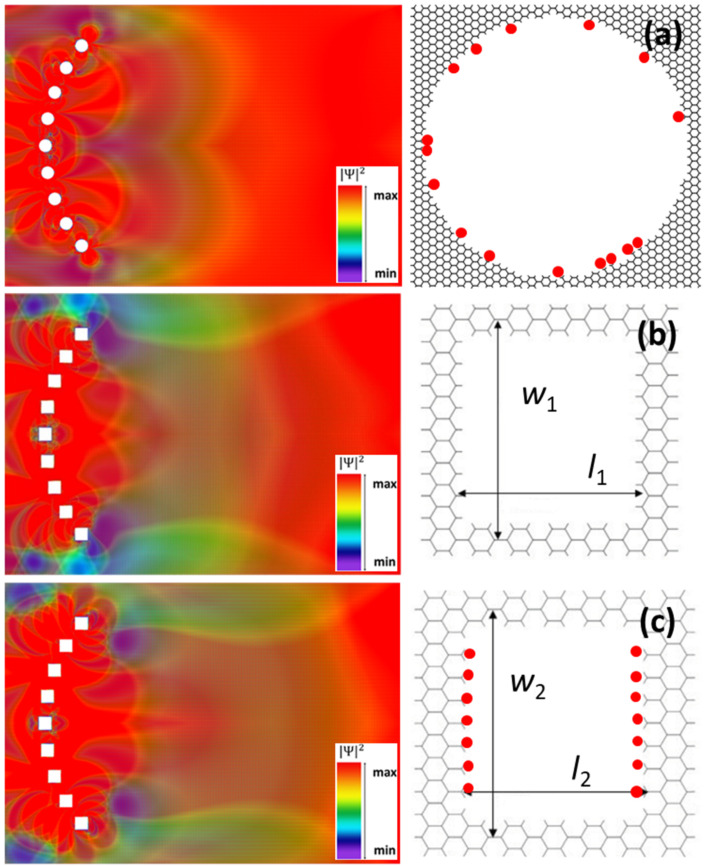
Spatial distribution of the square wavefunction modulus (in arbitrary units) for the *Pz* orbital in a GNR with an array of (**a**) circular and (**b**,**c**) square nanopores arranged on an arc of a circle.

We began our study by placing nine circular nanopores, with diameters of 8.362 nm, on an arc of a circle with a radius of 91.72 nm, subtending an angle of 80°; the angle between nanopores is thus 10°, as shown in Figure 1a. These circular pores were obtained by subtracting 2141 C atoms from the GNR; note that the C-C distance in graphene is 0.142 nm. The left side of Figure 1a presents the spatial distribution of the charge carrier probability/square modulus of the wavefunction across the GNR in this situation for *l* = 234.229 nm and *w* = 165.894 nm. The energy of charge carriers was chosen as *E* = 0.73 meV, value inside the interval ΔE = 0.51–5.2 meV, energy interval for which only one transverse mode exists in the GNR. For all visualizations in this paper, the color map is purple/blue for low values and red for high values of the wavefunction, the square modulus of which is represented in arbitrary units.

The right side of Figure 1a presents the detailed geometry of the circular pore, including its H terminations. We have highlighted with red dots the C atoms passivated with two H atoms. As follows from Figure 1a, electrons are mainly localized around nanopores and then found with equal probability at distances larger than the circle radius, no net focusing being observed. In fact, one can argue that a weak antifocusing can be observed along the propagation direction immediately after the nanopore array. The interference pattern in Figure 1a is the result of strong electron scattering by nanopores, the overall form of the spatial distribution of electron probability (not shown) being quite similar for different energy and pore size ranges, as long as the single-transverse-mode condition is fulfilled. This result, obtained despite the fact that the nanopores’ dimensions and configuration were chosen to agree/scale with the corresponding arrangement in the plasmonic lens, is a reminder of the differences between charge carriers in graphene and surface plasmon polariton excitation and propagation. 

Because the edges of nanopores are instrumental in defining the interference pattern of the electron wavefunction in the GNR, and the circular nanopores have mixed armchair and zig-zag edges, as well as mixed hydrogenation types, we investigated in the following a similar configuration containing square nanopores with the same sides as the circular ones arranged on the same arc of a circle. These have zig-zag edges along the width of the GNR and armchair edges along its length. The right sides of Figure 1b,c show a detailed view of the edges of nanopores (drawn intentionally smaller in order to highlight the edges). As can be seen from these figures, depending on the corners of the nanopores, two distinct configurations can be defined, with specific associated hydrogenation types, as determined by the relaxation condition of the structure: with one H atom in Figure 1b and with two H atoms along the transverse direction in Figure 1c, highlighted again with red circles. The C atoms along the GNR length are all terminated by one H atom. The dimensions of the nanopores are *w*_1_ = 8.874 nm, *l*_1_ = 8.538 nm (obtained by removing 2760 C atoms from the GNR), and *w*_2_ = 8.874 nm, *l*_2_ = 8.378 nm (corresponding to 2690 removed C atoms). In both cases, the corresponding spatial distribution of the electron probability, shown in the left sides of Figure 1b,c, respectively, is similar to that for the configuration with circular nanopores, the region of (weak) antifocusing behind the nanopore array being more extended.

**Figure 2:**
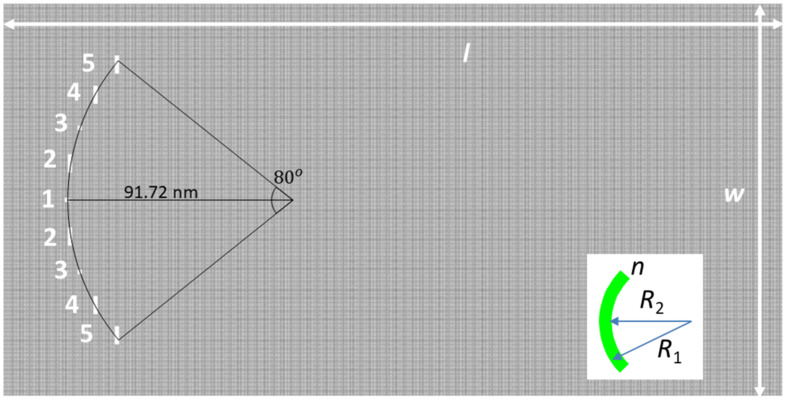
Schematic configuration of the GNR with the focusing/antifocusing nanopore array. Inset: equivalent optical-focusing meniscus with refractive index *n* and radii *R*_1_ and *R*_2_.

Although a weak antifocusing/antilocalization can be observed in the configurations in Figure 1, the effect is not strong enough. We have, therefore, considered further several geometries of rectangular nanopores with well-defined edge types (and hydrogenation) in order to maximize the phenomenon, and have identified the arrangement illustrated in Figure 2 as the one that maximizes the focusing/antifocusing effect. The geometry is similar to that considered previously, in the sense that there are nine nanopores with zig-zag edges along the width of the GNR and armchair edges along its length, arranged symmetrically on an arc of a circle with the same radius *r* = 91.72 nm, subtending an angle of 80°; the angle between nanopores is thus also of 10°, the distance between their centers being of 16 nm along the length of the arc. The difference with respect to the previously studied configurations is that the nanopores are no longer identical: the central one, numbered 1 in Figure 2, and the nanopores denoted by 3 have an almost square shape, while the nanopores labeled as 2, 4 and 5 have a rectangular shape. The center of the circular arrangement of nanopores is situated at 30 nm from the left side of the structure and at half width. Note that the widths of the nanopores along the propagation direction of charge carriers are significantly reduced, favoring wavefunction tunneling at the nanopores, and hence increased transmission. Only in this way can the phase information of the incident wavefunction, essential in determining constructive interferences responsible for focusing, be (at least in part) preserved; otherwise, as shown in the examples above, the wavefunction pattern is a result of strong scattering by the nanopores’ edges, independent of their shape. After reducing the dimensions of nanopores along the propagation axis to about one-fourth of the initial value, we analyzed arrays of nanopores with different dimensions along the transverse direction and with well-defined edge types. Several configurations with almost square and rectangular nanopores were investigated, the nanopores in the central area (nanopores 1 to 3) being found to have a higher degree of influence on the wavefunction distribution. Finally, we found that the nanopore array configuration in Figure 2 has the strongest effect on the spatial distribution of the wavefunction.

As in the previous case, we studied, separately, the two nanopore geometries drawn at the right sides of Figure 1b,c, and associated with different hydrogenation types transverse to the propagation direction (along *w*). The width and length of these two types of nanopores are labeled, as before, with *w*_1_, *l*_1_ and *w*_2_, *l*_2_. These two hydrogenation types affect the propagation of electrons rather differently, as detailed below, inducing either localization/focusing or antilocalization/antifocusing of the charge carrier wavefunction. 

### 3.1. Wavefunction Focusing/Localization by the Nanopore Array

Numerical simulations performed with NEMO5 showed that electron focusing can occur in structures with one-hydrogen terminated zig-zag nanopore edges (as in Figure 1b), with the following dimensions: *w*_1_ = 2.465 nm, *l*_1_ = 2.134 nm (153 C atoms removed from the GNR) for nanopores 1 and 3; and *w*_1_ = 8.874 nm, *l*_1_ = 2.134 nm (621 carbon atoms removed) for nanopores 2, 4 and 5. The GNR has dimensions *w* = 170.813 nm, *l* = 421.667 nm, only one transverse mode propagating for electron energies in the range ΔE = 2.57–4.2 meV.

**Figure 3:**
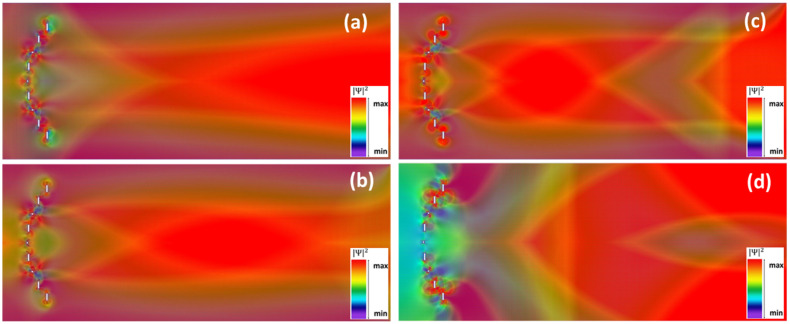
Spatial distribution of the square wavefunction modulus (in arbitrary units) for the *Pz* orbital in the GNR configuration in Figure 2 with single-hydrogenated nanopores for (**a**) *E* = 2.59 meV, (**b**) *E* = 3.39 meV, (**c**) *E* = 4.13 meV, and (**d**) *E* = 4.28 meV.

For this configuration, as can be seen from Figure 3, electron localization toward the center of the GNR/focusing occurs throughout the energy interval for one-mode propagation, the energies selected for emphasizing this effect being *E* = 2.59 meV (in Figure 3a, at the beginning of ΔE), *E* = 3.39 meV (in Figure 3b, at the middle of ΔE), and *E* = 4.13 meV (in Figure 3c, at the end of ΔE). The focal point (more specifically, the center of the focal region) shifts towards the nanopore array as the electron energy increases and a clear focalization region can be distinguished in Figure 3b,c; only half of the lenticular-focusing region is visible in Figure 3a due to the limited GNR length imposed by the memory of the simulation hardware. In addition, the focusing power increases/the charge carriers are localized in a smaller longitudinal region for higher *E* values.

This dependence of the focal length on the electron energy is in agreement with Snell’s law for charge carriers in graphene [3,21], for which the wavenumber 2*pn*/*l* of an optical field with wavelength λ in a medium with a refractive index *n* is replaced by $\left(E-V\right)/\hbar v_{F}$, where *V* is the potential energy in graphene and $\nu_{F}$ is the Fermi velocity. Consequently, since in classical optics the focal length of a lens with given radii of curvature is inversely proportional to *n* (more exactly, to n−1), in the case of the nanopore array in graphene the focalization power increases/the focal length decreases with increasing *E*. 

For still higher energies, outside the interval ΔE, the focusing effect is no longer observed. This fact can be seen in Figure 3d, where *E* = 4.28 meV; in this case, the spatial distribution of the square modulus of the wavefunction becomes more complex as the number of propagating modes increases. 

**Figure 4:**
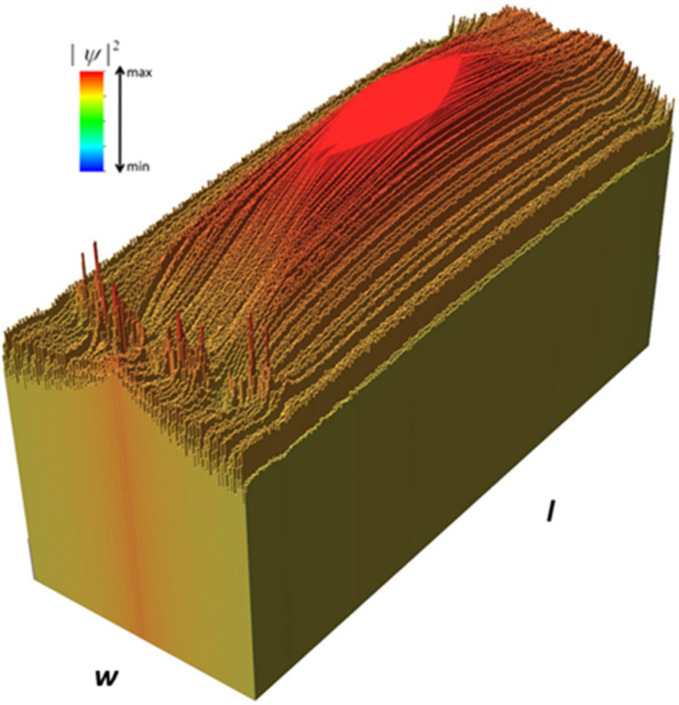
Histogram of the probability distribution for Figure 3b.

A more intuitive representation of the focusing effect is given in Figure 4, which presents a histogram of the probability distribution for *E* = 3.39 meV. The spikes at the bottom left of this figure are associated with the nanopore arrangement. Figure 4 also shows that in the focal region, colored red, the value of the square modulus of the wavefunction is about 1.2 times larger than at the edges of the nanoribbon. Although an enhancement of the electron probability takes place in the focal region, the probability distribution is rather flat across this area, so that no definite focal point can be identified. 

### 3.2. Wavefunction Antifocusing/Antilocalization by the Nanopore Array

The antifocusing effect occurs in GNRs with the second type of nanopore hydrogenation: zig-zag graphene edges (shown in Figure 1c) terminated with two hydrogen atoms. The considered structure has the following geometric parameters: *w* = 165.894 nm, *l* = 234.229 nm, with *w*_2_ = 2.465 nm, *l*_2_ = 1.992 nm (152 C atoms removed) for nanopores 1 and 3; and *w*_2_ = 8.874 nm, *l*_2_ = 1.992 nm for nanopores 2, 4 and 5 (obtained by removing 620 carbon atoms). In this case, numerical simulations performed with NEMO5 showed that there is only one transverse propagating mode for electron energies in the range ΔE = 0.51–5.2 meV.

**Figure 5:**
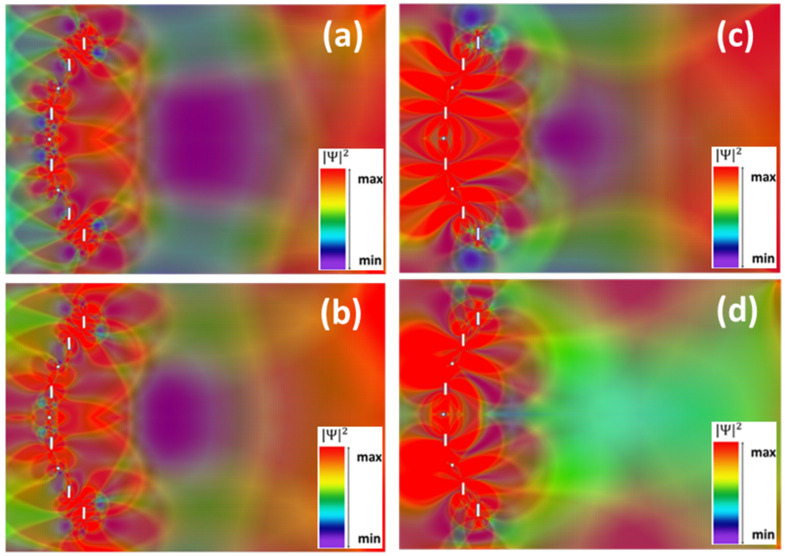
Spatial distribution of the square wavefunction modulus (in arbitrary units) for the *Pz* orbital in the GNR configuration in Figure 2, with double-hydrogenated nanopores for (**a**) *E* = 0.53 meV, (**b**) *E* = 0.73 meV, (**c**) *E* = 1.08 meV and (**d**) *E* = 1.60 meV.

As in the previous section, we investigated the spatial distribution of electron probabilities at different energy values, the results being presented in Figure 5a–d for *E* = 0.53 meV, 0.73 meV, 1.08 meV, and 1.6 meV, respectively. A shorter length *l* was chosen in Figure 5 in comparison with that in Figure 4 for a clearer representation of the antilocalization/antifocusing effect: the spatial distribution of the square modulus of the wavefunction is uniform at larger *l* values than those in Figure 5. As follows from this figure, antilocalization/antifocusing of charge carriers occurs for low energies (in Figure 5a–c), this effect disappearing for higher *E* values, still in the energy range of single-transverse-mode propagation. (Note that, while similar to Figure 1, this effect is more pronounced for the configuration studied here.) For low *E* values, the spatial extent of the antilocalization region along the electron propagation direction (along *l*) decreases as *E* increases and shifts slightly toward the nanopore array. This shift and the antifocalization power are similar to those in Figure 3, although no antifocusing system exists in classical optics. 

**Figure 6:**
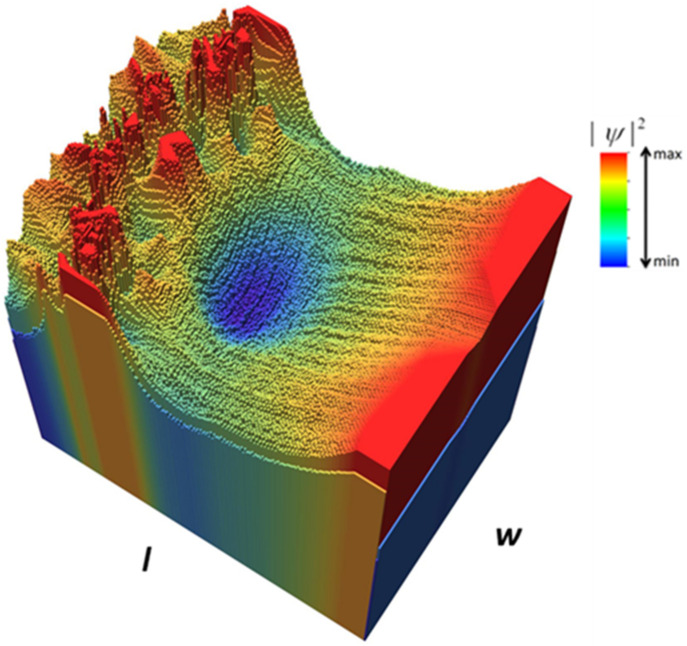
Histogram of the probability distribution for Figure 5b.

A histogram of the probability distribution for *E* = 0.73 meV is shown in Figure 6, the nanopore arrangement being correlated with the spikes visible at the top left of this figure. In the antifocusing region, represented in blue in Figure 6, the square modulus of the electron wavefunction is only 58% of the maximum value, attained in the region surrounding it and about 67% from the corresponding value at the edges of the nanoribbon; the antifocusing effect is thus more pronounced than focusing and, moreover, a clear and quite narrow region of minimum electron probability (basically, an antifocal point) can be identified. 

## 4. Discussions and Conclusions

Inspired by the analogies between the propagation of ballistic electrons and electromagnetic fields, we intended to expand this analogy to devices (in particular, a lens) relying on surface plasmon polaritons. The challenge was considerable, since the excitation of such plasmonic waves and the working principles of plasmonic-based devices are different from the counterparts involving non-surface-bound propagating electromagnetic waves. Although encountering difficulties, we managed to find a suitable configuration of rectangular nanopores, with well-defined hydrogenated edges, which focuses an incident ballistic electron wavefunction, the focal length depending on the electron energy. In the process we have also revealed the opposite effect: antifocusing of electron wavefunction, which has no counterpart in classical optics. 

The next step in our investigation was to determine the origin of the focusing/antifocusing phenomenon for electrons in graphene. The origin could not be similar to that for surface plasmon polaritons, namely constructive interferences of point sources determined by the holes arranged on an arc of a circle [22]. Because, unlike other electron lenses [5,15,17,18,19,20], in our proposed configuration there are no specifically shaped gate electrodes that allow the refraction of charge carriers, the focusing effect can only be explained using the analogy with an optical meniscus, as that illustrated in the inset of Figure 2. Such a meniscus, formed from a medium of refractive index *n* in air, and radii *R*_1_ = *r + dr*, *R*_2_ = *r* − *dr*, with *dr << r*, has a focal length *f* determined from the relation 1/*f* = (*n* − 1) (1/*R*_1_ − 1/*R*_2_) ≅ -(*n* − 1)2*dr*/*r*^2^. The array of nanopores can be seen as a non-perfect analogue of such a meniscus. More precisely, since the nanopores are subwavelength structures for the ballistic electrons in graphene (for *E* = 3 meV, the corresponding wavelength is about 1.5 μm), we can replace the circular area containing the nanopores with an equivalent, homogeneous medium which presents to the incoming electrons a potential barrier of height *V*. Therefore, knowing that the refractive index in optics is equivalent to $\left(E-V\right)/\hbar v_{F}$, in order to obtain our simulated results, i.e., *f* = 200 nm for *r* = 91.72 nm, 2*dr* = 2.5 nm, we would need a potential barrier height of about 11 meV. Whether for a meniscus-shaped area with *V* = 11 meV, the electrons would focus tightly or not, the potential energy distribution in the nanopore array is not uniform (the potential barriers are located only in the rectangular nanopores and not between them) and scattering events on the nanopore edges cannot be ignored, so that a non-perfect focusing is to be expected. 

Whereas focusing of the electron wavefunction, as in classical optics, can be associated with constructive interference, i.e., with increased electron localization, antifocusing in the nanopore array configuration could be regarded as weak localization, in particular as the counterpart of weak antilocalization (as opposed to localization) in nanodevices with randomly placed scattering centers [41]. As mentioned before, it is a phenomenon with no classical counterpart, and hence the formula used above to estimate the equivalent potential energy of the nanopore array region in this case has no significance. Still, as the effect takes place at a shorter distance to the nanopore arrays, it suggests an equivalent higher (at least in magnitude) potential barrier value of the nanopore array region (a formally negative *f* value—suggesting an opposite effect, not related to a divergent optical meniscus—corresponding to antifocusing at about 100 nm after the nanopore array would require a potential barrier of about −22 meV), which would enhance the contrast between the potential energy in the nanopore array region and surrounding graphene, and could explain the enhanced antifocusing effect.

It should be noted that such a large difference between the effect of the nanopore array in the focusing and antifocusing configurations cannot be attributed to the quite small difference in the dimensions of the pores (identical widths and only 7% difference in lengths between focusing and antifocusing configurations), but must be associated with the different hydrogenation types of the nanopore zig-zag edges, which can be controlled through the chemical potential of hydrogen [38]. Indeed, there are many references relating to the different behavior of single- and bi-hydrogenated zig-zag graphene nanoribbons with respect to edge formation [42], binding energy for the edge modification [43], or the possibility to form heterojunctions between graphene zig-zag nanoribbons with different hydrogenation types [44,45]. All the above-mentioned studies refer to graphene edges parallel to the transport direction of electrons, whereas in our case the graphene edges are normal to the electron flow direction; no studies of the last situation exist, to the best of our knowledge. Although our numerical approach could not, in principle, determine the heights of the potential barriers, the value of 11 meV as the average/equivalent potential energy of the nanopore array region, obtained from the quantum-classical analogy, is plausible. This issue deserves further attention, which is, however, not the aim of the present paper.

Regarding the feasibility of fabricating the graphene nanopore array, we mention first that there are established methods to synthesize [46,47] or cut [48] graphene edges with a well-defined type (armchair or zig-zag). The simulations in this paper have shown that the graphene nanopore array requires small, rectangular (with well-defined edge types) nanopores. Small nanopores, with 5 nm diameter or even less, compatible with the nanopore dimensions used in our simulations, can be fabricated with a wide range of physical and chemical methods [49], including a helium-ion microscope [50], or electrical pulses [51]. Although the large majority of studies regarding nanopore formation have concentrated on controlling the size of an otherwise circular pore, few can produce rectangular nanopores. In particular, arrays of rectangular nanopores have been synthesized with atomic precision in [52], whereas the atom-by-atom nucleation (by Ar+ ion bombardment, which produces 1–2 atom defects) and the growth method (using TEM) of graphene nanopores described in [53] could be used to fabricate rectangular nanopores if the scanning motion of the TEM is adequately modified; a similar procedure to fabricate subnanopores, which could be employed to synthesize rectangular nanopores, is described in [54]. Thus, the available technology could fabricate nanopore arrays in graphene with atomically precise shapes and dimensions.

Our results, based on numerical simulations, highlight both similarities and differences between the propagation and excitation of electrons and electromagnetic fields, in particular surface-bound plasmonic fields. As such, they could stimulate further studies in the fascinating field of quantum-classical analogies.

## Data Availability

The authors confirm that the data supporting the findings of this study were generated using the method, software and parameters indicated within the article.

## References

[1] Dragoman D., Dragoman M. (2004). Quantum-Classical Analogies.

[2] Henderson G.N., Gaylord T.K., Glytsis E.N. (1991). Ballistic electron transport in semiconductor heterostructures and its analogies in electromagnetic propagation in general dielectrics. Proc. IEEE.

[3] Dragoman D. (2010). Polarization optics analogy of quantum wavefunctions in graphene. J. Opt. Soc. Am. B.

[4] Mihalache I., Dragoman D. (2011). Graphene analogy to electromagnetic field propagation. J. Opt. Soc. Am. B.

[5] Cheianov V.V., Fal’ko V., Altshuler B.L. (2007). The focusing of electron flow and a Veselago lens in graphene p-n junctions. Science.

[6] Bliokh K.Y., Smirnova D., Nori F. (2015). Quantum spin Hall effect of light. Science.

[7] Schubert M., Kühne P., Darakchieva V., Hofmann T. (2016). Optical Hall effect—Model description: Tutorial. J. Opt. Soc. Am. A.

[8] Arteaga O., Garcia-Caurel E., Ossikovski R. (2019). Stern-Gerlach experiment with light: Separating photons by spin with the method of A. Fresnel. Opt. Express.

[9] Zhang W., Zhang X., Kartashov Y.V., Chen X., Ye F. (2018). Bloch oscillations in arrays of helical waveguides. Phys. Rev. A.

[10] Gozman M.I., Polishchuk Y.I., Polishchuk I.Y., Tsivkunova E.A. (2015). Anomalous optical Bloch oscillations. Solid State Commun..

[11] Cserti J., Pályi A., Péterfalvi C. (2007). Caustics due to a negative refractive index in circular graphene p–n junctions. Phys. Rev. Lett..

[12] Darancet P., Olevano V., Mayou D. (2009). Coherent electronic transport through graphene constriction: Subwavelength regime and optical analogy. Phys. Rev. Lett..

[13] Ochiai T., Onoda M. (2009). Photonic analog of graphene model and its extension: Dirac cone, symmetry, and edge states. Phys. Rev. B.

[14] Zandbergen S.R., De Dood M.J.A. (2010). Experimental observation of strong edge effects on the pseudodiffusive transport of light in photonic graphene. Phys. Rev. Lett..

[15] Spector J., Weiner J.S., Stormer H.L., Baldwin K.W., Pfeiffer L.N., West K.W., Bauer G., Kuchar F., Heinrich H. (1992). Ballistic Electron Optics, in Low-Dimensional Electronic Systems.

[16] Neto A.H.C., Guinea F., Peres N.M.R., Novoselov K.S., Geim A.K. (2009). The electronic properties of graphene. Rev. Mod. Phys..

[17] Yan C., Pepper M., See P., Farrer I., Ritchie D., Griffiths J. (2020). Demonstration of electron focusing using electronic lenses in low-dimensional system. Sci. Rep..

[18] Lee G.-H., Park G.-H., Lee H.-J. (2015). Observation of negative refraction of Dirac fermions in graphene. Nat. Phys..

[19] Tang Y., Cao X., Guo R., Zhang Y., Che Z., Yannick F.T., Zhang W., Du J. (2016). Flat-lens focusing of electron beams in graphene. Sci. Rep..

[20] Paredes-Rocha E., Betancur-Ocampo Y., Szpak N., Stegmann T. (2021). Gradient-index electron optics in graphene p−n junctions. Phys. Rev. B.

[21] Chen S., Han Z., Elahi M.M., Habib K.M.M., Wang L., Wen B., Gao Y., Taniguchi T., Watanabe K., Hone J. (2016). Electron optics with p-n junctions in ballistic graphene. Science.

[22] Yin L., Vlasko-Vlasov V.K., Pearson J., Hiller J.M., Hua J., Welp U., Brown D.E., Kimball C.W. (2005). Subwavelength focusing and guiding of surface plasmons. Nano Lett..

[23] Yang J., Ma M., Li L., Zhang Y., Huang W., Dong X. (2014). Graphene nanomesh: New versatile materials. Nanoscale.

[24] Dragoman M., Dinescu A., Dragoman D. (2018). Solving the graphene electronics conundrum: High mobility and high on-off ratio in graphene nanopatterned transistors. Physica E.

[25] Verschueren D.V., Yang W., Dekker C. (2018). Lithography-based fabrication of nanopore arrays in freestanding SiN and graphene membranes. Nanotechnology.

[26] Oh J., Yoo H., Choi J., Kim J.Y., Lee D.S., Kim M.J., Lee J.-C., Kim W.N., Grossman J.C., Park J.H. (2017). Significantly reduced thermal conductivity and enhanced thermoelectric properties of single- and bi-layer graphene nanomeshes with sub-10 nm neck-width. Nano Energy.

[27] Dragoman M., Dragoman D. (2008). Plasmonics: Applications to nanoscale terahertz and optical devices. Prog. Quantum Electron..

[28] Zhang J., Zhang L., Xu W. (2012). Surface plasmon polaritons: Physics and applications. J. Phys. D.

[29] Lent C.S., Kirkner D.J. (1990). The quantum transmitting boundary method. J. Appl. Phys..

[30] Frensley W.R., Frensley W.R., Einspruch N.G. (1994). Chapter 9. Quantum transport. Heterostructures and Quantum Devices.

[31] Fonseca J.E., Kubis T., Povolotskyi M., Novakovic B., Ajoy A., Hegde G., Ilatikhameneh H., Jiang Z., Sengupta P., Tan Y. (2013). Efficient and realistic device modeling from atomic detail to the nanoscale. J. Comput. Electron..

[32] Steiger S., Povolotskyi M., Park H., Kubis T., Klimeck G. (2011). NEMO5: A parallel multiscale nanoelectronics modeling tool. IEEE. Trans. Nanotechnol..

[33] Lake R., Klimeck G., Bowen R.C., Jovanovic D. (1997). Single and multiband modeling of quantum electron transport through layered semiconductor devices. J. Appl. Phys..

[34] He Y., Kubis T., Povolotskyi M., Fonseca J., Klimeck G. Quantum transport in NEMO5: Algorithm improvements and high performance implementation. Proceedings of the 2014 International Conference on Simulation of Semiconductor Processes and Devices (SISPAD).

[35] TAmeen A., Ilatikhameneh H., Klimeck G., Rahman R. (2016). Few-layer phosphorene: An ideal 2D material for tunnel transistors. Sci. Rep..

[36] Mladenovic D., Sandu T., Dragoman D. (2020). Electrical rectification in asymmetric graphene nanoribbons with pores. Physica E.

[37] Boykin T.B., Luisier M., Klimeck G., Jiang X., Kharche N., Zhou Y., Nayak S.K. (2011). Accurate six-band nearest-neighbor tight-binding model for the pi-bands of bulk graphene and graphene nanoribbons. J. Appl. Phys..

[38] Wassmann T., Seitsonen A.P., Saita A.M., Lazzeri M., Mauri F. (2008). Structure, stability, edge states, and aromaticity of graphene ribbons. Phys. Rev. Lett..

[39] Lu Y.H., Wu R.Q., Shen L., Yang M., Sha Z.D., Cai Y.Q., He P.M., Feng Y.P. (2009). Effects of edge passivation by hydrogen on electronic structure or armchair graphene nanoribbon and band gap engineering. Appl. Phys. Lett..

[40] Li J., Sanz S., Castro-Esteban J., Vilas-Varela M., Friedrich N., Frederiksen T., Peña D., Pascual J.I. (2020). Uncovering the triplet ground state of triangular graphene nanoflakes engineered with atomic precision on a metal surface. Phys. Rev. Lett..

[41] Tikhonenko F.V., Kozikov A.A., Savchenko A.K., Gorbachev R.V. (2009). Transition between electron localization and antilocalization in graphene. Phys. Rev. Lett..

[42] Fujita N., Hasnip P.J., Probert M.I.J., Yuan J. (2015). Theoretical study of core-loss electron energy = loss spectroscopy at graphene nanoribbon edges. J. Phys. Condens. Matter.

[43] Lee G., Cho K. (2009). Electronic structures of zigzag graphene nanoribbons with edge hydrogenation and oxidation. Phys. Rev. B.

[44] Cao C., Chen L.-N., Long M.-Q., Xu H. (2013). Rectifying performance in zigzag graphene nanoribbon heterojunctions with different edge hydrogenation. Phys. Lett. A.

[45] Zeng J., Chen K.-Q., He J., Zhang X.-J., Sun C.Q. (2011). Edge-hydrogenation-induced spin-filtering and rectifying behaviors in the graphene nanoribbon heterojunctions. J. Phys. Chem. C.

[46] Talirz L., Ruffieux P., Fasel R. (2016). On-surface synthesis of atomically precise graphene nanoribbons. Adv. Mater..

[47] Yamada Y., Kawai M., Yorimitsu H., Otsuka S., Takanashi M., Sato S. (2018). Carbon materials with zigzag and armchair edges. ACS Appl. Mater. Interfaces.

[48] Ci L., Xu Z., Wang L., Gao W., Ding F., Kelly K.F., Yakobson B.I., Ajayan P.M. (2008). Controlled nanocutting of graphene. Nano Res..

[49] Yang T., Lin H., Zheng X., Loh K.P., Jia B. (2017). Tailoring pores in graphene-based materials: From generation to applications. J. Mater. Chem. A.

[50] Deng Y., Huang Q., Zhao Y., Zhou D., Ying C., Wang D. (2017). Precise fabrication of a 5 nm graphene nanopore with a helium ion microscope for biomolecule detection. Nanotechnology.

[51] Kuan A.T., Lu B., Xie P., Szalay T., Golovchenko J.A. (2015). Electrical pulse fabrication of graphene nanopores in electrolyte solution. Appl. Phys. Lett..

[52] Moreno C., Vilas-Varela M., Kretz B., Garcia-Lekue A., Costache M.V., Paradinas M., Panighel M., Ceballos G., Valenzuela S.O., Peña D. (2018). Bottom-up synthesis of multifunctional nanoporous graphene. Science.

[53] Russo C.J., Golovchenko J.A. (2012). Atom-by-atom nucleation and growth of graphene nanopores. Proc. Natl. Acad. Sci. USA.

[54] Su S., Xue J. (2021). Facile fabrication of subnanopores in graphene under ion irradiation: Molecular dynamics simulations. ACS Appl. Mater. Interfaces.

